# Plant Classification from Bat-Like Echolocation Signals

**DOI:** 10.1371/journal.pcbi.1000032

**Published:** 2008-03-21

**Authors:** Yossi Yovel, Matthias Otto Franz, Peter Stilz, Hans-Ulrich Schnitzler

**Affiliations:** 1 Animal Physiology, Zoological Institute, University of Tuebingen, Tuebingen, Germany; 2 Max-Planck-Institute for Biological Cybernetics, Tuebingen, Germany; 3 University of Applied Sciences, Konstanz, Germany; University of California San Diego, United States of America

## Abstract

Classification of plants according to their echoes is an elementary component of bat behavior that plays an important role in spatial orientation and food acquisition. Vegetation echoes are, however, highly complex stochastic signals: from an acoustical point of view, a plant can be thought of as a three-dimensional array of leaves reflecting the emitted bat call. The received echo is therefore a superposition of many reflections. In this work we suggest that the classification of these echoes might not be such a troublesome routine for bats as formerly thought. We present a rather simple approach to classifying signals from a large database of plant echoes that were created by ensonifying plants with a frequency-modulated bat-like ultrasonic pulse. Our algorithm uses the spectrogram of a single echo from which it only uses features that are undoubtedly accessible to bats. We used a standard machine learning algorithm (SVM) to automatically extract suitable linear combinations of time and frequency cues from the spectrograms such that classification with high accuracy is enabled. This demonstrates that ultrasonic echoes are highly informative about the species membership of an ensonified plant, and that this information can be extracted with rather simple, biologically plausible analysis. Thus, our findings provide a new explanatory basis for the poorly understood observed abilities of bats in classifying vegetation and other complex objects.

## Author Summary

Bats are able to classify plants using echolocation. They emit ultrasonic signals and can recognize the plant according to the echo returning from it. This ability assists them in many of their daily activities, like finding food sources associated with certain plants or using landmarks for navigation or homing. The echoes created by plants are highly complex signals, combining together all the reflections from the many leaves that a plant contains. Classifying plants or other complex objects is therefore considered a troublesome task and we are far from understanding how bats do it. In this work, we suggest a simple algorithm for classifying plants according to their echoes. Our algorithm is able to classify with high accuracy plant echoes created by a sonar head that simulates a typical frequency-modulated bat's emitting receiving parameters. Our results suggest that plant classification might be easier than formerly considered. It gives us some hints as to which features might be most suitable for the bats, and it opens possibilities for future behavioral experiments to compare its performance with that of the bats.

## Introduction

When orienting in space and searching for food, microchiropteran bats continuously emit echolocation signals. The returning echoes are analyzed in the auditory system to perform the basic echolocation tasks of detection, localization and classification [1]. Classification of vegetation probably plays a major role in spatial orientation and in food acquisition. It is fundamental for recognizing landmarks and vegetation edges which are mandatory for the route following behavior observed in bats [2]. In addition it is also very important for finding and recognizing foraging habitats such as meadows, bushes, trees etc. which are indicators of specific food sources [3],[4]. In all of these cases the vegetation has to be classified from a relative long distance of up to a few meters. The behavior of bats in the field indicates that bats notice background structures within the so called edge space which extends up to around 6 m [5]. It has also been shown that Natterer's bats learn to discriminate conifers from broad-leaved trees and that horseshoe bats commuting along a hedge of bushes show distinct reactions in their echolocation behavior when the reflection properties of the bushes are changed by covering them with velvet (Denziger and Schnitzler, unpublished data). In addition to the classification of vegetation types, bats can also identify parts of plants like flowers and fruits. Glossophagine bats for instance, find new nectar sources by classifying the shape and texture of flower echoes [6],[7].

Plants have complex shapes that cannot be described in terms of simple geometrical primitives [8]. From an acoustical point of view, a plant can be approximated as a stochastic array of reflectors formed by its leaves. McKerrow et al. [9] removed the leaves from pot plants and discovered that the contribution of branches to the echoes is minor. In large plants the stem might also play a role. In broad-leaved plants, the reflectors are relatively flat and usually large compared to the emitted wavelengths (∼0.3–1.5 cm) in a typical frequency modulated bat call. Hence, the backscatter from a broad-leaved plant typically is a superposition of reflections, with statistics determined by the characteristics of the foliage such as the size and the orientation of the leaves, along with their spatial distribution. The overall duration of the echoes is a result of these parameters too. In dense foliage, for instance, surface leaves will acoustically shadow deeper ones, thus strongly attenuating the sound waves that penetrate beyond the outer surface. These properties also apply to conifer trees, except for the fact that they possess needle-shaped reflectors that are small relative to a considerable part of the emitted wavelengths. Conifers are therefore regarded as diffuse scatterers that produce many small echo components, whereas broad-leaved plants lead to pronounced amplitude peaks in the echoes, referred to as glints.

Although the importance of classifying complex objects is well discussed in the scientific bat literature, very little is known about how bats actually perform classification. Only a few previous studies directly addressed the question of object classification using echolocation in bats, and most of them did so in the context of classifying objects with rather simple shapes [10],[11],[12], or only a few reflectors [13],[14]. The few experiments that tested the bat's ability to classify relatively complex echoes [15],[16] did not suggest an explicit mechanism to explain it. The studies that examined classification of simple objects usually assumed simple cues that could be easily recognized in the temporal, frequency or time-frequency representation of the echoes as a basis for classification such as, for instance, a certain notch arrangement in the frequency domain. This approach is hardly feasible for real plant echoes due to their complexity and the strong dependency on the angle of acquisition which makes the ad hoc identification of such features a difficult task. Another typical approach is to identify peaks corresponding to reflections from parts of the object and to compare them to stored echoes that represent known objects or known geometrical shapes (e.g., edges, corners and surfaces). The comparison can be done by measuring the difference between the echoes directly [15] or by comparing certain representative statistics [17]. Once again, these methods will face severe difficulties with complex echoes, mainly since the echoes returning from different reflectors always highly overlap and are very hard to isolate. A few studies trying to classify complex echoes such as vegetation echoes [13],[18] and Stilz and Schnitzler unpublished data relied on extracting one or several parameters (e.g. peak intensity, average intensity and etc.) from some representation of the echoes, with a subsequent selection of those parameters that best assign the plant echoes to their corresponding classes. Thus, the set of all tested parameters is determined by the experimenter beforehand. This has advantages and disadvantages: on the one hand, parameters are usually chosen according to physical or biological plausibility which simplifies their interpretation, but on the other hand strong assumptions are made by choosing a fixed set of candidate parameters since some of the important features might be overlooked.

In this paper, we propose a new approach to complex echo classification. We use a linear classification technique that comes originally from the field of machine learning. We use this technique to operate directly on the raw spectrogram magnitude of the echoes, without the intermediate step of specifying some set of potentially relevant parameters or features. With this approach we take advantage of the statistical structure of the data itself in order to identify the best features to classify it. Thus, the technique allows for the exploration of a wide range of features simultaneously, and often finds simple ones. This comes at the price that the obtained results are slightly harder to interpret on first sight, but we will provide a thorough analysis of the features that are extracted from the data. Our classifiers are trained on a large database of natural plant echoes, created with a bat-like ultrasonic frequency modulated signal. We show that the trained classifiers are able to classify echoes from previously unseen plants with high accuracy. At the same time, our method provides a systematic analysis of all linear features in the echo spectrograms of the database in terms of their relevance for classifying the underlying plant species. More over our approach enables classification of vegetation echoes using a single echo. This coincides with recent work [14] that showed that bats can classify a complex 3D object using a single ensonifying position, without the need to integrate the information from echoes over different acquisition angles. The presented approach provides many insights regarding the task of plant echo classification and is sufficiently general to be applied to other types of complex echoes, for instance from food sources or landmarks.

## Results

### General Results

A linear SVM classifier is able to distinguish between any of the five tested plant species and any other species or group of species, based on a comparison between two single echoes, one from each class. For the classification task of discriminating one species from the rest already a simple linear classifier achieves very high percentage of discrimination (80–97%, see Table 1 for details). The classification of spruce or corn from the other species is almost perfect whereas the classification of the three broad-leaved trees, and especially the beech, from the rest was the most difficult. For the pairwise classification (Table 2) the relatively poor result for the classification of beech vs. blackthorn, both broad-leaved trees, stands out. The relatively high standard deviation in this case implies that a larger data set might improve performance. Comparing the task of pairwise classification in general to the task of one species vs. the rest reveals that the latter is the more difficult one. This is expected since a group of species always contains much more intrinsic variation that the classifier has to learn, but even with this difficulty, our linear classifiers performed surprisingly well. In the next sections we will mainly discuss the task of classifying one species against the rest, except for cases in which the pairwise comparison reveals more interesting phenomena.

**Table 1:** Area under the ROC curve for the five classification tasks of one species vs. the other four.

apple	spruce	blackthorn	beech	corn field
0.88 (0.04)	0.97 (0.02)	0.91 (0.04)	0.81 (0.05)	0.95 (0.02)

The standard deviations are computed from a five-fold cross validation. Classification performance of the one species vs. the rest task.

**Table 2:** Area under the ROC curve for the ten classification tasks of one species vs. another one.

Species	spruce	bk. thorn	r. beech	corn field
apple	0.99 (0.01)	0.93 (0.02)	0.90 (0.03)	0.98 (0.01)
spruce	*	0.98 (0.03)	0.99 (0.01)	0.98 (0.02)
bk. thorn	*	*	0.90 (0.07)	0.98 (0.02)
r. beech	*	*	*	0.95 (0.03)

The standard deviations are computed from a five-fold cross validation.

Classification performance of the pairwise task.

### The Decision Echo

**Figure 1:**
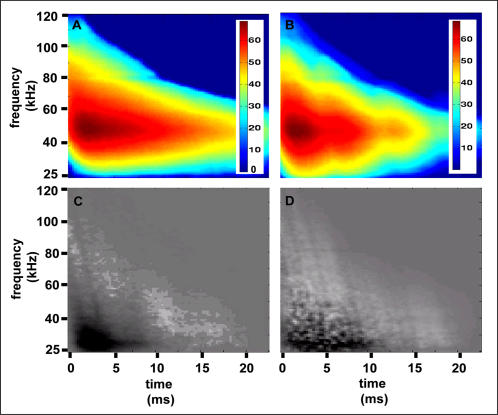
Decision echo analysis for the classification task of spruce vs. the rest. (A) Average spectrogram of the raw data of spruce. (B) Average spectrogram of the raw data of all the plants except spruce (i.e. the rest). The color bars for both (A) and (B) are in dB. (C) The difference of the preprocessed spectrograms of spruce and the rest. (D) The normal vector (decision echo) to the separating hyperplane calculated for this classification task. In both (C) and (D) black represents negative values, white represents positive ones, and gray is zero.

**Figure 2:**
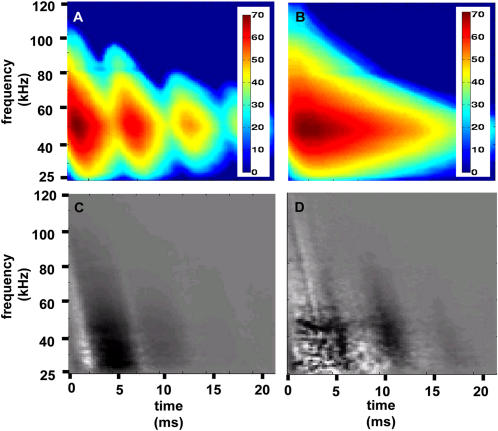
Decision echo analysis for the classification task: corn vs. the rest. (A) Average spectrogram of the raw data of corn. The color bars for both (A) and (B) are in dB. (B) Average spectrogram of the raw data of all the plants except corn (i.e. the rest). (C) The difference of the preprocessed spectrograms of spruce and the rest. (D) The normal vector (decision echo) to the separating hyperplane calculated for this classification task. In both (C) and (D) black represents negative values, white represents positive ones, and gray is zero.

The weights of the normal vector to the separating hyperplane 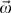, i.e., the decision echo, has the same dimensionality as the data, and can assist in better understanding the features that are used by our machines for classification. Since we are using linear machines, the class of an echo is actually determined by the sign of the inner product of the preprocessed echo and the decision echo, after adding the offset. This means that the regions of the decision echo that have high absolute (depicted dark or bright in the figures) values have more influence on the decision. In order to interpret the decision echo, we present the decision echoes of the classification tasks of spruce vs. the rest and corn vs. the rest together aside an image of the difference between the average spectrograms of the two classes (Figures 1 and 2). Comparing the decision echoes and the spectrogram differences (Figures 1C and 1D, 2C and 2D) it becomes clear that in both classification tasks our classifiers are actually emphasizing the areas in which the differences between the spectrograms are most salient. The comparison of the differences between the decision echoes of the two tasks shows that in the task of classifying spruce from the rest, the classifier performs a combination of a frequency domain analysis and a time domain analysis. In the early parts of this task's decision echo, low frequencies are inhibitory (with negative values) while the high frequencies are excitatory (with positive values). In the later parts (∼ after 10 ms) the entire decision echo is excitatory (excluding regions with larger attenuation as will be explained below). Therefore, classification of spruce can be generally described as a measurement of the difference between the high and low frequencies intensities in the spectrogram's early parts (frequency domain analysis) and as a measurement of all intensities in the later parts (time domain analysis). The classification of the corn field is mainly a time domain analysis. Here the regions in the decision echo which are compatible with the first and second rows of the field (compare with the corn spectrogram in Figure 2A) are excitatory, while the gaps between these rows are inhibitory. The effect of the frequency dependent atmospheric attenuation of sound waves is expressed in all of the decision echoes. According to this attenuation, the higher the frequency of the wave is, the faster its intensity decreases with the distance. This gives the decision echoes a triangular shape, meaning that the higher the frequency, the less the later parts of the spectrograms are used for classification (gray regions in Figures 1 and 2).

### Generation of Artificial Hybrid Spectrograms and Echoes

**Figure 3:**
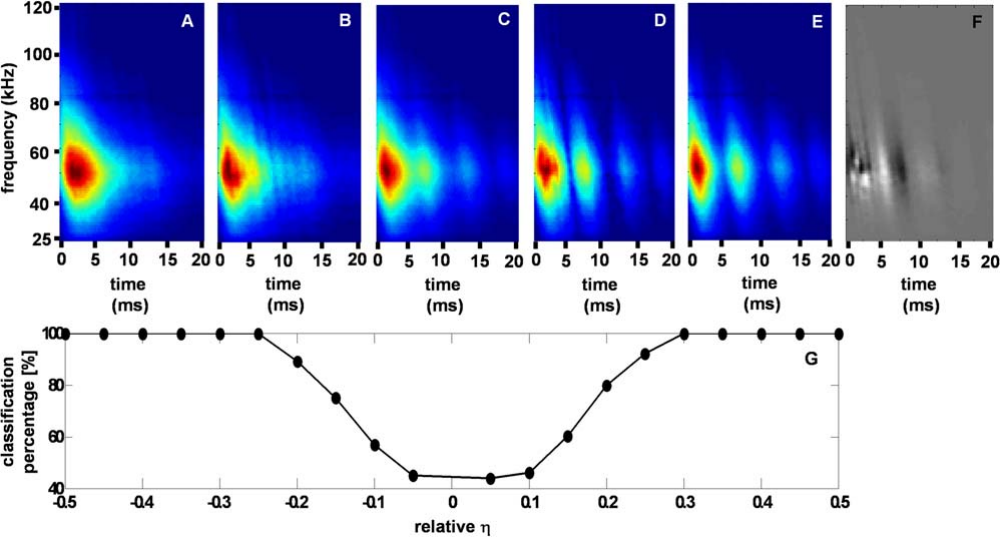
The results of generating hybrid sepctrograms of apple and corn. Only (B) and (D) were artificially generated. Color bars are not presented, but the data are in the spectral power scale. (A) Average spectrogram of apple. (B) The decision echo multiplied by η = 0.07 added to the average spectrogram. (C) The average spectrogram of corn and apple. (D) Same as B, but with η = −0.07. (E) Average spectrogram of corn. (F) The decision echo calculated for this task used to create (B) and (D). Dark intensities depict negative values, while white depict positive ones. (G) Classification performance of echoes created from artificial hybridized spectrograms as a function of the η factor. To measure performance we divided the spectrograms of each species into 10 groups, each containing 50 spectrograms with a similar η. The units of η are relative, such that η = 1 corresponds to an artificial spectrogram that is as distant to the hyperplane as the most distant original spectrogram. The performance is measured in the percentages of echoes that were correctly classified according to the expected classification.

An alternative interpretation of the decision echo is the direction in the high-dimensional input space along which the changes between the two classes are maximal. In other words, for a pair of species it represents the transition between the two. Inspired by Macke et al. we calculated for each pair of species the average spectrogram, and then added the decision echo multiplied by a positive or negative factor η. By doing this we actually move along the direction of the maximum change from a mean representation of the two plants in the directions of each one of them. We used this method to generate 1000 artificial spectrograms that are hybrids of different ratios of the apple vs. corn pair (500 on each side of the hyperplane see Figure 3).

To generate echoes from the hybrid spectrogram, we propose to use the random phase method described in the Materials and Methods section. We did so in order to verify our method, and the resulting echoes lead to a consistent classification behavior, i.e., higher classification performance for larger absolute values of η (see Figure 3 for more details)

### Support Vectors

**Figure 4:**
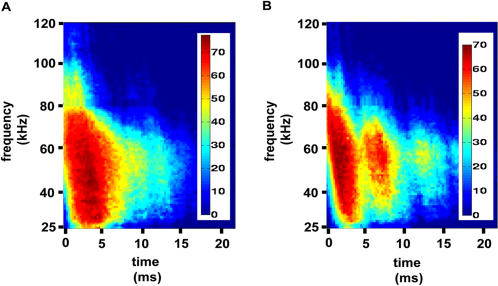
Spectrograms of the weighted support vectors on each side of the hyperplane. The color bars are in dB. (A) The apple spectrograms used as support vectors added up according to their weights. (B) Same as A for corn. Examining the two weighted spectrograms, the idea of the support vectors, being the most difficult data points to separate in the limits of the data set, becomes clearer.

To determine the separating hyperplane, the SVM uses only a limited number of data points (the ones that are closest to the hyperplane) which are termed support vectors. The importance of the *i*th support vector is weighted by a constant α*_i_*. Adding up the support vectors on each side of the hyperplane separately, with the proper weighting, provides another view on the classification rule. For an arbitrary pair of two species, a weighted sum of the support vectors on one side of the hyperplane can be intuitively understood as the most similar this species can acoustically be to its pair in the limits of our data set. The spectrograms of the weighted support vectors for the pair of apple tree and corn field reveals how in some cases an apple tree can acoustically resemble a corn field and vise versa (Figure 4).

### Frequency vs. Time Information

**Figure 5:**
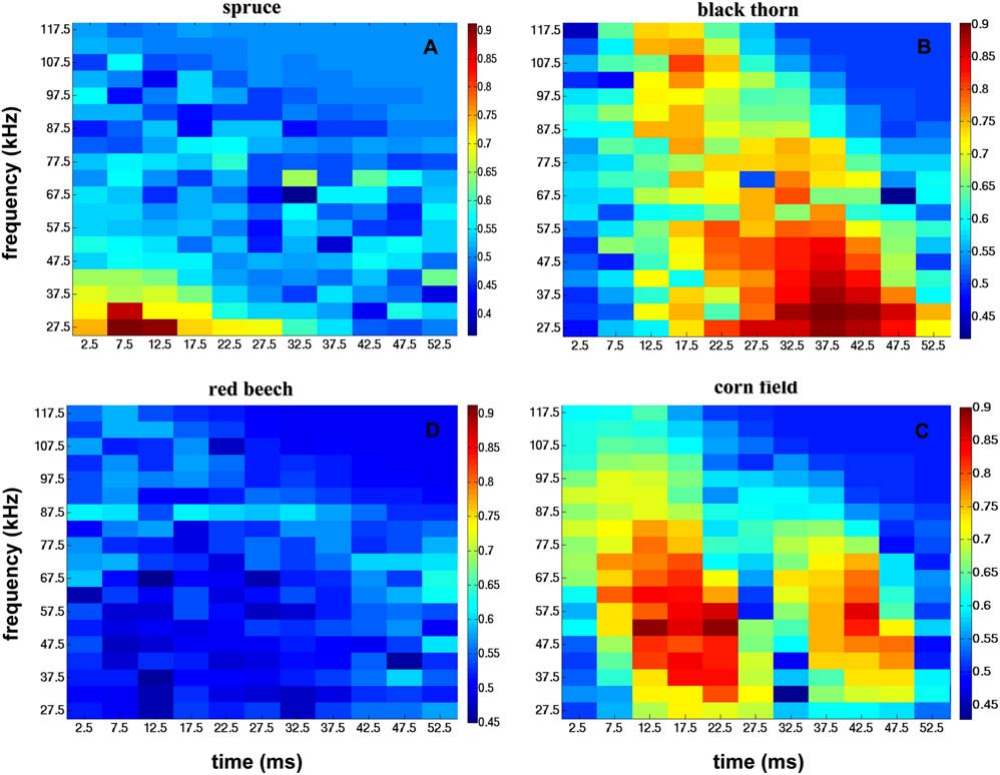
Classification performance of four classification tasks when using partial data of the spectrograms for classification. Each pixel represents the performance when using a square from the spectrogram with a frequency band of 5 kHz and time duration of 5 ms. The color denotes the area under the ROC curve (AUC) when classifying using only this square of information from the spectrograms. The classification tasks presented are: (A) Spruce vs. the rest; (B) Blackthorn vs. the rest; (C) Beech vs. the rest; (D) Corn field vs. the rest.

From the decision echoes we learned that both time and frequency information are used for classification and that in higher frequencies the earlier parts of the spectrograms are preferred for classification, probably due to atmospheric attenuation. Here we test whether classification is possible when only parts of the spectrogram's information are used. We divided the spectrograms into squares of 5 kHz by 5 ms, and for each square, we trained and tested SVMs for all the classification tasks in the same manner described above. We found that already the information contained in one of the limited squares within the spectrogram is sufficient for classification with very high (∼0.9) performance in all cases except for beech (Figure 5). However, the exact position of this limited sensitive region in the time-frequency space can be significantly different for different classification tasks. In spruce classification for instance the low frequencies in the beginning of the echo provide the best classification performance. In blackthorn on the other hand the later parts of the spectrogram are better for classification, and there is a wide range of frequencies and times that can be used with almost equal performance.

### Generalization over Different Angles

Our classifiers generalized over different aspect angles. This can already be learnt from the basic experiments since we trained them by using data from all angles, and then tested them with high success on data from all angles (Tables 1,2). In a different version of the one species vs. the rest experiment we trained machines using training data recorded from all angles except for the tested one and then tested on data points from only the tested angle. The classification performance in these experiments stayed as high as in the ones in which data from all angles were used to train and test the machines with no significant difference (Two way ANOVA, F_2,60_>0.86, P<0.45).

### The Effects of Preprocessing on Performance

In order to examine the sensitivity of the performance of our machines to the preprocessing of the data, we used a cross-validation approach to estimate the performance while changing the parameters of the preprocessing steps. This was done on the training data set as explained in the methods section for two procedures: the effect of cutting out the echoes in the time domain, and the effect of the time-frequency resolution (i.e., the DFT window length used to calculate the spectrogram).

To test the effect of cutting the echo out in the time domain, we changed the threshold according to which the cutting points were determined. Cutting the echo improved the classification performance by a non significant average of 0.02 (Two way ANOVA, F_2,60_>1.78, P<0.18) We attribute this slight improvement to the registering effect that this procedure has on the echoes. Applying a threshold is closely equivalent to recognizing the first wave front of the echoes and this aligns them before any further processing. The two different cutting criteria (10 or 20 times above noise level) showed no difference what so ever.

**Figure 6:**
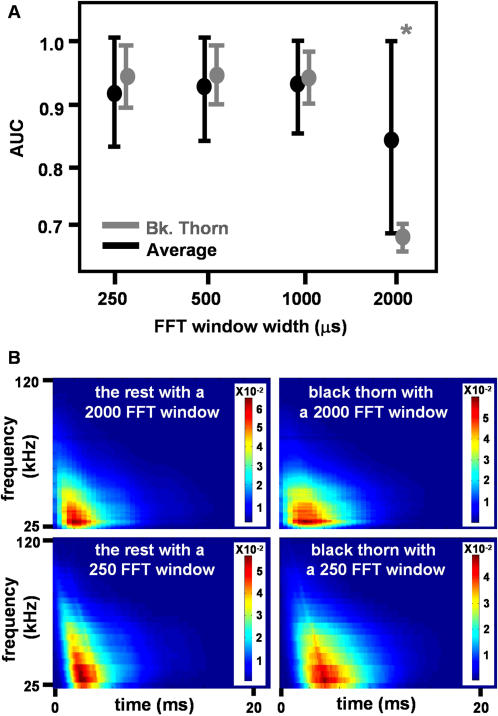
Effect of the DFT window length on classification performance. (A) The area under the ROC curve (AUC) for four different window lengths ranging from 250–2000 µs. Average results are presented together with the blackthorn classification case, in which the effect was most clear. The difference between a 2000 µs window length and the other lengths is significant (P<0.05), whereas the difference between the three other lengths is not. (B) Average spectrograms for a window length of 2000 µs (first row) and a 250 µs one (second row) for the classification task of blackthorn vs. the rest. It can be seen how time information is decreased (i.e. smeared) for the 2000 µs window (first row). This makes separation between the two classes easier with the 250 µs window (second row) even when only examining them visually.

To determine the effect of the DFT window length we varied it and kept the percentage of the overlap between sequential windows constant (Figure 6). The extent of the spectrograms in the temporal direction decreased with window length whereas the extent in frequency increased such that the overall information remained constant. Up to a certain window length (1000), representing a time bin of 1ms (with 80% overlap) the window length had no significant influence on classification performance. Above this length however, for the 2000 window, there was an overall significant decrease (0.07 on average) in classification performance (2-way ANOVA, F_3,80_>18.5, P<0.0001). This decrease mainly affected the three classification tasks blackthorn vs. rest (0.25 on average, 1-way ANOVA, F_3,16_>24.8, P<3^−6^), beech vs. rest (0.13 on average, 1-way ANOVA, F_3,16_>6.5, P<0.005) and corn vs. rest (0.03 by average, 1-way ANOVA, F_3,16_>2.85, P<0.07) while the performance of the other two tasks did not change. The decrease is probably a result of the loss of time information due to excessive smoothing. In general, the most suitable window length depends on the specific classification task.

## Discussion

### General Conclusions

In this work we analyzed the characteristics of a database containing vegetation backscatter from five plant species ensonified with a bat-like ultrasonic pulse from different aspect angles. We used a linear classification technique to find discriminative features in the backscatter spectrograms that were able to differentiate between different plant species independent of aspect angle. In contrast to previous approaches, we did not derive these features from biological or practical plausibility assumptions. Instead, discriminative features were *learned* from the statistical regularities found in our database. When we tested our classifiers on a single echo from a new, previously unseen specimen from one of the species in the database, classification performance was surprisingly high, ranging between 0.8–0.99. This indicates that the echoes created by a frequency modulated ultrasonic sweep can be highly informative about the plant's species membership. This forms a possible explanatory basis for some of the observed abilities of bats in classifying complex objects such as landmarks or vegetation as indicator for food sources [3],[4].

Once a linear classifier is trained, it can also be used as a generative model. This means that the learnt features can be used to generate new artificial examples of the data. In our case we could create new echoes of a certain plant species or of a combination of species (Figure 3). In the future we hope to use this type of artificially generated echoes in behavioral experiments in order to test the correlation between our linear functions and bat classification performance.

### What Did the Classifiers Actually Learn?

**Figure 7:**
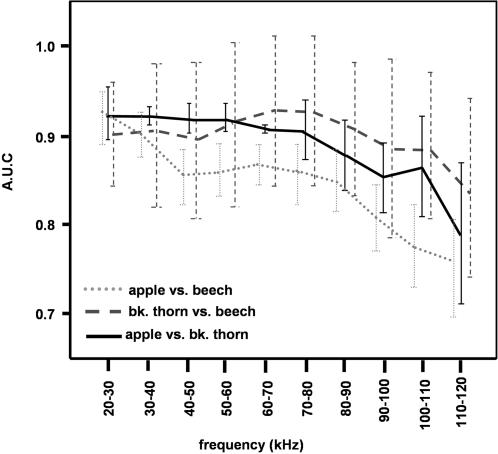
The area under the ROC curve (AUC) for all of the broad-leaved trees pair-wise classification, when using partial information from the spectrograms, limited to frequency bands of 10 kHz. The graphs show a relative preference for the low frequencies information, but the exact slope is task-specific.

As described in the methods, we designed our preprocessing procedure in such a way as to minimize the species-specific noise (due to external or internal recording parameters) to prevent the classifiers from using it for classification. The probability that such artifacts still retain some influence on our results is quite low considering the actual information that leads to a classification decision as depicted in the decision echoes. All decision echoes (see examples in Figures 6 and 7) give a higher weight to regions of the spectrogram where the signal of at least one of the classes is high above the noise level. Regions with lower signal intensities, i.e. later in time and higher in frequency, tend to have values close to zero in the decision echoes. As an additional test, we repeated the same classification experiments, but this time after preprocessing the echoes with a Wiener filter [19], which uses the noise spectrum in order to filter out the noise from the entire signal, not only from the low amplitude regions. The noise spectrum for each echo was estimated in the same way as described in the methods. There was no significant difference in the classification performance of the classifiers with and without Wiener denoising (F_1,48_>1.6, P<0.22). The results after denoising appear to be slightly (but not significantly) better which implies that the measurement noise does not contain species-specific artifacts that could be erroneously used by the algorithm for classification. When examining the decision echoes it seems that some of them (e.g. corn classifiers, see Figure 2) use the time structure of the echoes more than the frequency content, while others (e.g. spruce classifiers, see Figure 1) use the frequency content more than the time structure. In general, in all cases both time and frequency information was used for classification. Regarding the best features of the plants used for classification, it seems that our classifiers neither use the overall extent, nor the fine texture of the spectrogram. Instead they rely on intermediate scale structures, such as the representative frequency content in a certain time interval or a characteristic time structure for certain frequencies. In most cases we could identify a small region in the spectrogram which is already sufficient for classification. However, the exact position of this decisive region in the time-frequency plane can significantly change between the different classification tasks. This means that if nothing is known about the classified plant species beforehand, a large proportion of the spectrogram is required to achieve a good performance over all tasks. Thus, a call with a large frequency bandwidth, as is observed in frequency modulating bats, is preferable from the classification point of view.

**Figure 8:**
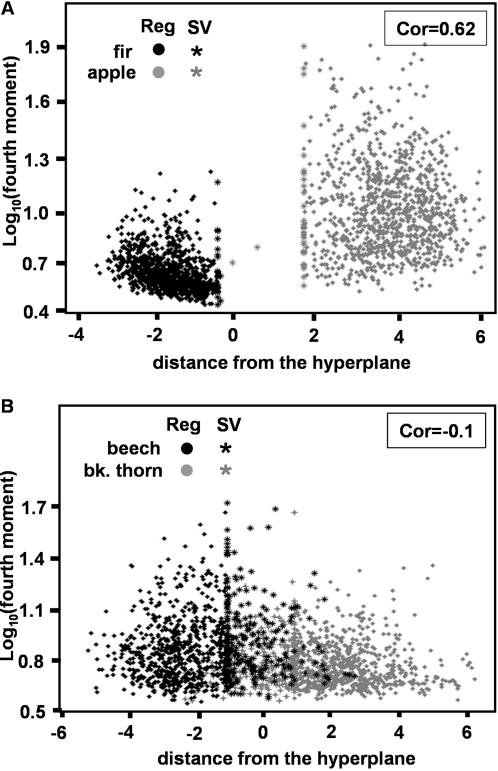
The correlation between the distance from the separating hyperplane and the fourth moment of the echoes. o – regular data point, * – support vectors. Correlation values are indicated in rectangles in upper right corner. (A) The comparison for the task of classifying apple and spruce reveals a high correlation between the distance and the fourth moment. (B) The comparison for the task of classifying beech and blackthorn reveals no correlation between the distance and the fourth moment, implying that the fourth moment cannot be used to classify the two. This figure also visualizes how the task in (A) is easy for the SVM compared to the one (B).

A plant is a complex object comprised of many reflectors (mainly the leaves). Although the spatial arrangement of the different plant species contributes to the echo structure, it can be helpful to regard the plant leaves as an array of independent, rather simple reflectors to understand the differences in the frequency content of species. In our study we found that the most suitable frequencies for classification are not necessarily the ones with the best signal to noise ratio (SNR). The highest SNR was usually attained around 50 kHz, whereas the frequencies with the best classification performance were in most cases lower, indicating that the echoes vary more in the lower frequency range between species.

Some reason for these preferred frequency bands can be found in radar theory [20]. The cross section of a reflector depends on the geometry of the reflector in relation to the wavelength of the sound pulse. For a simple spherical reflector, the intensity of the echo depends on the ratio between the sphere's circumference and the wavelength of the emitted signal. This ratio defines three regions: (1) The Rayleigh region - if the circumference is smaller than the wavelength the intensity of the reflections decreases rapidly when decreasing the radius of the sphere. (2) The resonance region - if the wavelength is of the same order as the circumference (up to ∼10 times larger) the intensity of the reflection oscillates depending on the ratio mentioned above. (3) The optic region - if the circumference is much larger than the wavelength the intensity of the reflection is equal in all frequencies. This division into three domains exists also in reflectors with a more complex shape, but then the cross section will also depend on the angle of ensonification. The borders of these regions when considering the extreme frequencies of our emitted signal (25 and 120 kHz) are such that reflectors larger than 14 cm will be in the optic region for all frequencies, and reflectors smaller than 0.03 cm will be in the Rayleigh region for all frequencies. The reflectors in between will be in all three regions depending on the frequency. From the point of view of classification, it is clear that the Rayleigh region is the most advantageous since at a given frequency, the intensity of the reflection changes with the circumference, therefore providing direct information about the reflectors size. Clearly, this presupposes that the intensity is high enough to be perceived. The optic region on the other extreme provides no frequency information that could be used for classification, since the reflections in all frequencies are redundant. Obviously, the time structure can still be different. The resonance region shows a more complex interdependence between frequency and reflector size than both extremes, but a suitable classifier might be able to use this information.

In order to relate this theoretical framework to our data, we have to provide some approximation of our reflector's circumference. This is not easy, for the leaves on plants comprise of a range of many sizes, and they are not simple spheres. In the case of spruce, its needles prevent us from doing this, but it is safe to assume that it's very small radial dimension (up to a few millimeters) is equivalent to relatively high frequencies, above 100 kHz, and therefore most of its reflectors will behave according to the Rayleigh domain. Corn leaves on the other extreme are very long, and will therefore probably mainly behave according to the optic domain. As for the three broad-leaved trees, we use the roughly approximated average leaf length (calculated by measuring a variety of leaves) in order to estimate the relevant wavelength range. Apple and beech trees exhibit the largest leaves among the three, with an average length of around 8 cm. This is equivalent to a wavelength of a few kHz. Its reflectors should therefore behave according to the resonance domain when the emitted signals have frequencies of up to a few dozens of kHz, and according to the optic domain with higher frequencies. Blackthorn trees exhibit smaller leaves, with an average length of about 3 cm. This is equivalent to a wavelength of roughly 10 kHz, resulting in its reflectors being in the resonance domain for most of the frequencies of the signals emitted in this research.

Spruce classification is probably easiest to explain by to this approach. Its many reflectors in the Rayleigh region result in lower intensities in the low frequencies of its echoes (Figure 2). This means that it can be well classified by its lack of low frequency content. Indeed, as can be seen in the decision echo and time-frequency classification performance (Figures 3C and 7A), the information in low frequencies provide the best classification performance for spruce.

Corn field in contrast should not contain much frequency information, and truly its decision echo doesn't seem to be using any obvious frequency information (Figure 2D), and so does the time-frequency classification performance graph imply (Figure 5D).

In the case of the three broad-leaved trees (apple, beech and blackthorn) the effects of frequency are less obvious. We therefore examined the classification performance of each pair when only using parts of the spectrograms with a limited bandwidth of 10 kHz while retaining the entire time information. For all pairs, classification was best at low frequencies (Figure 7). For beech vs. blackthorn and apple vs. blackthorn, all frequency bands between 25–80 kHz lead to a similar classification performance, whereas in beech vs. apple, performance begins to drop already at the 30–40 kHz band. These could be explained by the above argumentation: all three plants exhibit leave sizes in a considerable large range such that for our emitted call all three species probably have reflectors both in the resonance and in the optic regions. Apple and beech trees, however, have bigger leaves than blackthorn and thus should have more reflectors in the optic region and less in the resonance region, particularly at higher frequencies. As a consequence, apple and beech should be harder to discriminate in this frequency range.

### Are the Extracted Discriminative Features Available to the Bat Brain?

Since the intent of our study is to test which features of plants echoes might enable bats to classify the plants, we have to examine if the information used by our classifiers is – at least in principle – available to the bat brain.

After the preprocessing of the received echoes our classifiers were trained to recognize plant species based on the magnitude of their spectrograms. This information is easily accessible to the bats through the spectro-temporal decomposition of the echo in the cochlea [21]. We ignored the phase information which to date has not unequivocally been proven to be used by bats. We also did not cross-correlate the recorded echoes with the emitted signal. This is often done in echolocation studies, thus revealing the impulse response (IR) of the ensonified object, although it is not known whether bats can actually use the IR. Finally, we use a time resolution of about 1ms which is far above the minimum time resolution which has been reported for bats [22],[23]. Thus it seems highly probable that the information used by our classifier is available to bats. Experimental evidence suggests that bats can extract information with a much higher resolution than required (see [23] for a summary).

### Do the Results Extend to More General Natural Scenes?

The classifiers were able to classify a plant correctly at acquisition angles that were not present in the training set, i.e., our classifiers generalize to a certain degree over the angle of acquisition. This result was unexpected, since in acoustics, as opposed to vision, a slight change of the acquisition angle can result in a very large change in the echo, as has been shown for plants [9],[11]. However, we noted above that our classifiers use intermediate-scale features which probably vary more slowly over the angle of acquisition. Moreover, most of the species in our database contain leaves in all orientations such that the local statistics do not change significantly with acquisition angle, even when the individual echoes vary considerably.

An issue that was not tested in this work is the generalization over distance, i.e. the ability to use the same classifiers on objects that were ensonified from different distances. The two main limiting factors regarding this generalization are the attenuation of the echoes and the change of the beam width. The attenuation affects the echoes in two ways: 1) The SNR of the entire echo deteriorates, in a frequency dependent manner. 2) The geometric attenuation increases with the square of the distance, and therefore the attenuation rate within the echo will change when it returns from different distances. The first problem of the overall SNR could be dealt with, up to a limit, by increasing the intensity of the emitted signal. In addition, our classifiers do not require the fine texture of the spectrograms for classification, and therefore can probably tolerate a certain deterioration of the SNR without a significant drop in performance. The second problem could be overcome – at least in principle – by using the absolute distance as measured by the arrival time of the echo to compensate for the attenuation differences within the echo.

As for the beam, its width will widen the further the emitter is from the plant, thus increasing the ensonified region. The larger the emitter distance, the more reflectors will contribute to the echoes. Taking into account the intermediate features used by our classifiers, we hypothesize that as long as our beam is wide enough to capture them, classification performance will stay high. A too wide beam, however, could introduce new echoes from other reflectors, which leads to a smearing effect due to the arrival of more reflections at close instants in time, and thus to a slow deterioration of classification performance. Although bat beams are usually much wider than the one used by us, it is clear that there exists a distance range in which the echo statistics are similar to our setting.

### Relation to Behavioral Studies

In one of the few reported works dealing with the bat's ability to classify complex echoes, Grunwald et al. [14] found that bats can distinguish the fourth moment of artificially created echoes. They conclude that bats might be using the changes in the fourth moment to facilitate navigation guided by echolocation. We tested this conclusion in the light of our results for two pair-wise classification tasks. To this end we calculated the fourth moment of each echo and compared it to its distance from the hyperplane (see methods). The results (Figure 8) show that in the rather simple task of classifying a conifer tree (spruce) from a broad-leaved tree (apple) the distance from the hyperplane of each echo is linearly correlated with its fourth moment (R∼ = 0.64, P<0.00001). However, since we were using only linear machines, our classifiers have no access to higher order statistics such as the fourth moment. This means that information sufficient to classify the two trees is also available in the low order statistics of the echoes. In the case of a difficult classification task (blackthorn vs. beech) on the other hand, we found a close to zero linear correlation between the distance from the hyperplane of the echo and its fourth moment (R∼ = 0.1, P<0.00001). Moreover when examining the data (Figure 8B) it is obvious that only the fourth moment is not a sufficient statistic for discriminating between these two broad-leaved tree species. In contrast, the SVM is able to find features that are sufficient for reliable classification of this pair already by relying on simple first- and second-order statistics.

Wichmann et al. have shown the relevance of a hyperplane calculated from the data to human categorization performance [24],[25]. They compared SVM-based classification with human performance on a task of image gender classification, and found that SVMs are able to capture some of the essential characteristics used by humans for classification. Furthermore, Wichmann and Macke were able to show that the distance from the separating hyperplane could be used to predict the certainty with which these decisions are made. Despite the fact that it is known that the brain can perform classification of nonlinear data, these works always used linear machines just as we did. In the future we would like to use the SVM as echo generators in order to test the relevance of our calculated hyperplanes to performance of the bat brain.

### Final Conclusion

We have found that the highly complex echoes created by ensonifying plants with a frequency modulated bat like signal contain vast species specific information that is sufficient for their classification with high accuracy. From the point of view of a bat, we prove that it can use a single echo received by one ear, with a surprisingly simple receiver, having a relatively low time resolution and no access to the impulse response, to extract the information required for classification. We also demonstrate how it can then apply a basic linear hyperplane that could be easily implemented by a neuronal apparatus, in order to classify the vegetation echoes. These findings could explain some of the abilities observed in natural bat behavior such as using landmarks for navigation, and finding food sources on specific vegetation.

## Materials and Methods

### Data Acquisition

**Figure 9:**
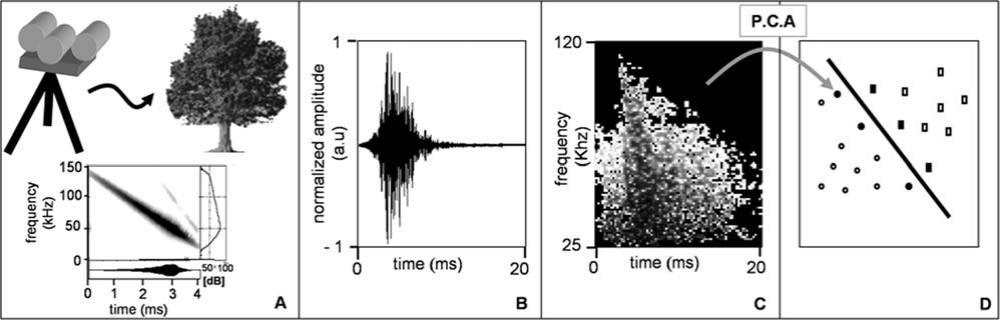
Summary of the materials and methods. (A) The basic setup of the experiments, in which a sonar head on a tripod was used to ensonify plants. The emitted signal's spectrogram is presented with the time signal under it and the frequency dependent intensity curve on the right. (B) An example of a time domain back scatter recorded from a single apple tree. The amplitude is in arbitrary units. (C) The spectrogram of the time domain signal of B, created after cutting the echo out of the time signal. The spectrogram's frequency range was cut between 120–25 kHz, and it was threshold leaving only the regions that are high above noise. (D) An illustration of the classification by SVMs. Following PCA, each spectrogram is represented by a 250-dimentional data point (shown in the figure as a 2-dimentinal point) belonging to one of two classes (circles or rectangles). The SVM then learns the best hyperplane for the training data. The data points that are closest to the hyperplane (denoted as full shapes) are called the support vectors and define the orientation of the hyperplane.

A biomimetic sonar system consisting of a sonar head with three transducers (Polaroid 600 Series; 4-cm-diam circular aperture) connected to a computer system was used to create and record vegetation echoes. The sonar head was mounted on a portable tripod. Its central transducer served as an emitter (simulating the bat's mouth) and the two side transducers functioned as receivers (simulating the ears). Backscatter received from the emitted signal was amplified, A/D converted, and recorded by a computer. The emitted signal resembles a typical frequency modulated bat call in terms of its duration and frequency content (Figure 9A). It comprises a four millisecond linear down-sweep from 140 to 25 kHz. We excited the emitter with a constant amplitude, but due to the speakers frequency response an uni-modal response function was created with a maximum around 50 kHz, providing an intensity of 112 dB (SPL) at the maximal frequency in a distance of 1m from the emitter. Most of the signal energy was contained in the frequency band between 25–120 kHz. The combined frequency response of our emitter and receivers resulted in a frequency response that resembles the one of a typical frequency modulated bat call. In contrast to bats our emitted sound pulse had a rather narrow beam width, with its first null for 50 kHz occurring around 15°, much lower than known for bat calls [26].

The recorded back scatter or echo (both terms will be equally used in this paper, Figure 9B) was digitized at a sampling rate of 1 MHz and with a 12-bit resolution. The length of the recorded echo was very long (40 ms corresponding to 6.8 m). It included a long tail of noise after the part with echoes returning from the target. This enabled exact estimation of the noise for each recording.

All recordings were performed in the field with real plants as targets. Five plant species were chosen, representing a variety of the common species available in the local bats environment. The species were:

- Apple tree (*Malus sylvestris*) – This species has large leaves, in a spacious arrangement. The trees were covered with fruit.
- Norway spruce tree (*Picea abies*) – This was the only conifer tree that was ensonified. Its branches are spread homogenously and evenly covered with needles. Will be referred to as spruce throughout the paper.
- Common beech tree (*Fagus sylvatica*) – This species is characterized by large flat leaves that are on each branch usually arranged in the same plane. Will be referred to as beech throughout the paper.
- Blackthorn tree (*Prunus spinosa*) – This species has smaller leaves than the other broad-leaved trees, without any specific orientation. This species was usually found in a formation of a hedge rather then as a single tree like the other trees.
- Corn field (*Zea mays*) – Whole fields of each specimen were ensonified exhibiting a typical row structure.

50 specimens of each species were ensonified, each one from 25 different aspect angles on an equally spaced 5×5 grid centered at the horizon and the midline of the tree. This was done by starting at the top most left point on the grid, 10 degrees above the horizon and 10 degrees left to its midline and then turning the sonar head right in sequential steps of 5 degrees along the 5 points of the first row. Next the head was lowered by 5 degrees and the procedure was repeated, this time towards the right. This procedure provided 1250 echoes for each species from each ear. The distance between plant and tripod was always 1.5 m, and the height of the tripod above ground was set to 1.35 m. The acquisition of data from different angles enabled us to test for the ability to identify species independent of the aspect angle. This is commonly done in image classification research [27], in which images of the same object are taken from different angles in order to test view point invariance. All of the signal processing was performed with matlab 7.0

### Signal Preprocessing

The recorded echoes went through several three preprocessing steps.

**Figure 10:**
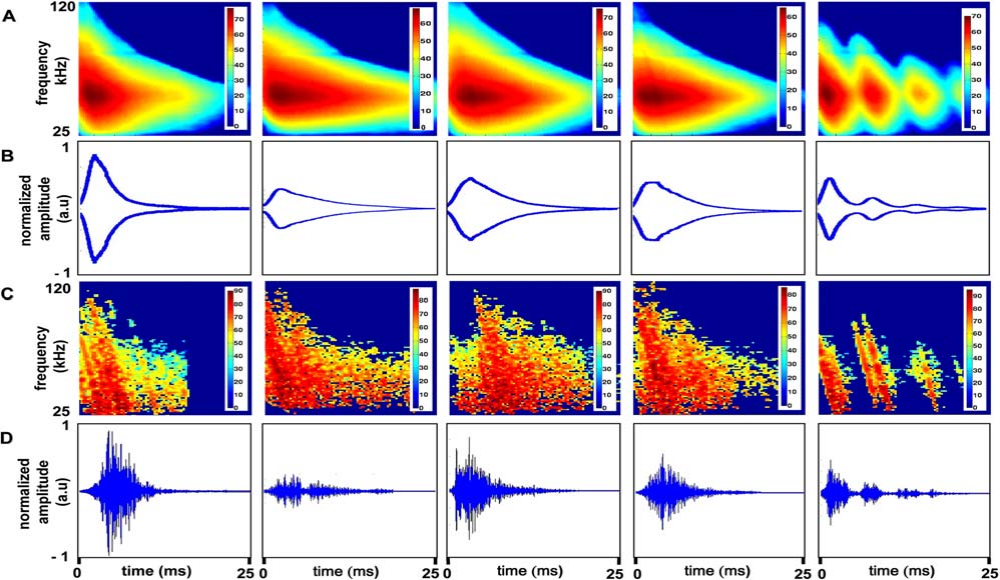
Raw data after preprocessing. In all rows the species from left to right are: apple, spruce, blackthorn, beech, and corn field. In all spectrograms, color bars are in dB. The units in the time signals are arbitrary. (A) The average spectrogram of each plant species. (B) The average envelope of the time signal of each plant species. (C) The corresponding example of a single spectrogram of each plant species (the effect of applying the threshold is noticeable). (D) The corresponding example of a single echo of each tree in the time domain.

1. In the first step the echo regions were cut out from the recorded signal in the time domain. For each recorded signal we estimated its noise level, using the last 5000 time samples of the signal. We then cut out the back scatter region or echo defined by the points in time for which the signal exceeded a preset threshold for the first and last time. The echoes between these two time points remained unchanged. The most suitable threshold above noise was found by using a cross-validation approach (see below). The cutting procedure was used to identify the first and the last wave front of each echo train, and so ensured that any further analysis of an echo will start at the first wave front and end with the last one. As a result of this step the echo differed in their duration, so we zero-padded their terminal part to match them to the longest one.
2. The next step transferred the cut echoes from the time domain into the time-frequency space by calculating the magnitude of their spectrograms (Figure 9C). We chose to perform the subsequent analysis in the time-frequency space both for technical considerations and from a biological point of view. On the technical side only this domain enabled us to simultaneously investigate the information contained both in time and in frequency domains. In addition, previous work showed that the time-frequency representation gives better object recognition performance [28]. From a biological point of view there are many models that describe the filtering activity of the auditory system; almost all are based on some form of time-frequency decomposition of the signal [21]. Instead of committing to one of these models we preferred to use the raw time-frequency data to avoid the possible information loss due to any specific model assumptions.
  The spectrograms were calculated with a Hann window and an 80% overlap between sequential windows. The window length of the DFT, and therefore the time-frequency resolution was treated as a free parameter that had to be determined. The most suitable length was found by using a cross validation approach (see below). The performance for various window lengths is presented in the results section. Unless stated otherwise, the results shown in the figures or discussed in the text, were created with a window length of exactly 1000 points, therefore providing a 1 ms time resolution (smoothed by the overlap) and a 1 kHz frequency resolution. We cut the spectrogram's frequency range so that it contained only the region of the emitted frequencies main intensity (i.e., 25–120 kHz). Through the remainder of the text we shall use the term spectrogram to describe the magnitude of the spectrogram.
3. The next step was intended to reduce the noise, and to avoid possible classification artifacts. This issue is not trivial, since the recordings of different plant species differed in their noise characteristics. There are many reasons for these species-specific noise characteristic. The recording of different species on different days can result in temperature variations of the environment which in turn leads to a different atmospheric attenuation. The varying recording locations can create a species-specific background noise. The noise characteristics also depend on the recording parameters, since two of the plants were recorded with a gain that was 2.5 times lower than the other three. Indeed a control experiment showed that a classification above chance level was possible by using spectrogram regions that contained only noise. The first noise reducing step was actually obtained in the first preprocess described above of cutting out the echo in the time domain. By doing this we ensured that only the parts of the echo that had a certain level above the noise went through any following analysis. We now aimed to exclude noise regions from the spectrograms frequency-time domain. To do so we computed the magnitude of the spectrogram of the noise signal of each echo (using the last 5000 time samples of the signal). We then selected for every spectrogram the maximum noise intensity at each frequency, thus calculating the maximum noise spectrum. This maximum noise spectrum was used as a threshold. For each time bin (i.e. column of the spectrogram) we set to zero any pixel of the spectrogram that was lower than five times the value of the maximum noise spectrum at that particular frequency. This procedure actually zeroed major parts of the spectrogram, thus ensuring that our classifier was only using the parts of the echo that were significantly above the noise level. For further comments regarding classification according to noise see the discussion section. Figure 10 shows examples of acquired data after the preprocessing.

### Machine Learning Computation and Preprocessing

For all training experiments described in the following paragraphs, the data was divided into a training (four fifth) and a test set (one fifth). This was done such that all the angular echoes of a specific plant individual were attributed either to the test or to the training set, but never to both, to prevent leakage of information from the test set to the training set, which might result in an overestimation of the generalization performance.

We performed two kinds of classification experiments. The first was a pairwise classification in which we trained ten machines, to distinguish between any possible pair of species. In the second, we trained five machines, each capable of distinguishing between one species and the other four. It should be mentioned that our classifiers categorize the plant using only a single echo. This is different from all the previous plant echo classification studies.

### PCA

After applying the above preprocessing methods, with a DFT window of 1000, each echo was represented by a 95 (frequency bins)×90 (time bins) = 8550-dimensional spectrogram, assuming here that the 1000 point window was used. Next each spectrogram was rearranged as a 8550-dimensional vector (simply by concatenating its columns) which left us with a total of 6250 echoes, each represented by a 8550-dimensional vector. We used Principle Component Analysis (PCA) to reduce the dimensionality of the data before applying the machine learning algorithms. We did this by projecting each data vector on the 250 eigenvectors with the highest eigenvalues. In every experiment, the eigenvectors were calculated for the covariance matrix of the training set exclusively. As a common PCA pre-process all 8550-dimensinal data vectors were first normalized to have equal energy. The PCA transformation reduced the dimensionality of the data so that each echo could now be represented by a 250-dimensional vector. The number 250 was another free parameter that was chosen via cross-validation (see below).

### Classification by Support Vector Machines (SVM)

We used linear Support Vector Machines (SVM, [29],[30], Figure 9D) as our classification algorithm. To implement the SVM we used the free “spider” software (http://www.kyb.mpg.de/bs/people/spider). An SVM is a state-of-the-art learning algorithm based on statistical learning theory. A linear SVM can be intuitively interpreted in a geometrical way as a separating hyperplane that divides the data set into two classes by minimizing the classification error of a training set and at the same time by maximizing its distance from the data points that are closest to it (Figure 9D). The hyperplane is simply a multidimensional plane that has the same dimensionality as the data points which correspond, in our case, to the spectrograms of the echoes after the above preprocessing. In many cases a perfect separation of the data into two classes is not possible due to outliers, or due to an overlap of the classes. Therefore the learning algorithm is adjusted to enable a certain amount of misclassified points. For this purpose a new constant C is introduced, that defines the penalty for misclassified points. This constant is known as the free parameter of the SVM - and as the other free parameters - it was determined by cross validation.

After training the SVM, classification was performed according to the following calculation:
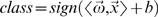
where 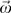 is a vector normal to the hyperplane 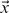 is a test echo (after preprocessing) and b is an offset (also calculated by the learning algorithm). The offset is equivalent to the free parameter in a three dimensional plane, and changing it moves the hyperplane along its normal direction When the result is +1 the echo will be classified as belonging to one species or species group and when it is −1 it will be classified as belonging to the other species or species group. It should be noted that SVMs is a non parametric method that makes no prior assumptions on the data and learns the classification rule using the data itself.

### Interpretation of the Results

The normal vector of the hyperplane is a weighted linear combination of the training data points:
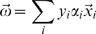
(2)Where *y_i_* is the sign (±1) attributed to each training data point according to its class label. The weights α*_i_* are a result of the learning procedure, and for most points they will be zero. Only the points that are closest to the hyperplane on both sides are assigned non-zero weights. These points are called support vectors, and actually define the orientation of the hyperplane. They can be interpreted as the most difficult points to separate in the limits of the data set. In visual classification studies the normal vector 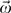 is interpreted as the decision-image [24],[25], so we will call it in our context the ***decision-echo***. The decision-echo can assist in better understanding the features that are used by the machine for classification. It has the same dimensionality of the data points after preprocessing, and since we were only using linear machines, the class of an echo is actually determined according to the sign of the inner product of the echo and the decision echo added to the offset. This means that the regions of the decision echo that have high absolute (non zero) values are more important for classification. An alternative interpretation for this vector is the direction along which the change between the two classes is maximal.

In addition to classification, one can calculate for each echo its distance from the hyperplane by:
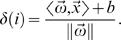
(3)


This measurement provides additional information regarding the ordering of our data points according to the classifier and can be used for further understanding of our performance.

### Model Selection by Cross-Validation

The four parameters of our model (i.e., the threshold above noise for the cut in time domain, the DFT window length, the number of principal components for projection and the C parameter of the SVM) were all determined using a five-fold cross validation. This means that for each possible value of the parameters, the training data set is divided into five sets of equal size, and each set serves as a test set for a classifier trained with this specific value on the other four sets. The value yielding the highest average classification rate was then chosen (see performance measurement below). It is important to note that this procedure was executed exclusively on the training set.

The first parameter – the threshold above noise level (step 1 of preprocessing) was determined independently of the other three, after they were already set. For this parameter the values 1, 10 and 20 times above noise level were tested.

The latter three parameters were determined via a cross validation on a 3-dimensional grid of parameter combinations. This means that for each possible combination of the free parameters on the grid the cross validation procedure was executed. The combination yielding the highest average classification rate was then selected. The possible values for these three parameters were as following: In the case of the window length the values 250, 500, 1000 and 2000 were tested. For the dimensionality reduction via PCA we tested the values 150, 200, 250 and 300 principle components and the values for C were evenly chosen on a logarithmic scale between 1 and 100000. For both the C parameter and the number of principle components the different parameters did not change the results significantly. The best parameters were 250 principle components and C = 10. The results for the best values for the DFT window length and the time domain threshold parameters are presented in detail in the results section.

### Performance Measurement

We also used a five-fold cross validation approach to test for possible overfitting of the classifiers, i.e over adjustment of the classifiers to the specific training sets in a way that does not represent the actual real world data. To do this we divided the entire data (i.e. not only the training set) into five equal sized parts each containing a training set (four fifth) and a test set (one fifth) in the same way that was described above. For each of these five parts the entire process of finding the best parameters was executed on the training set and the performance was then tested on the relative test set. This procedure created the standard deviations of the performance measures that are presented in the results section.

We used the area under the Receiver operating characteristic (ROC) curve to measure the performance of our classifying machines. The ROC curve is commonly used in psychophysics to estimate performance while changing a parameter. It is created by plotting the true positive rate (TP) on the Y axis and the false positive rate (FP) on the X axis, while changing a parameter. In our case the parameter along which TP and FP were plotted is the offset b of the hyperplane. Varying the offset is equivalent to moving the hyperplane along its normal direction (in parallel to itself). It is obvious that on one extreme case the rate of true and false positives will both be zero, and on the other extreme they will both be 1. Calculating the area under the ROC curves (depict as the AUC) evaluates the performance for all possible settings of b. The area ranges between 0.5–1, where 0.5 means a random classifier, and 1 means a perfect one. Any other value can be interpreted as the probability of ranking a positive data point higher than a negative one in a randomly drawn pair from the test data set. The standard deviations of the performance values were calculated for the results of the five different cross validation folds.

In order to compare classification performance of machines trained under different conditions (for instance when changing one of the above parameters), the classification performance measures were first transformed using the arcsin transformation:
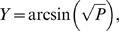
(4)where P is the area under the ROC curve for a certain classification task. The transformed data was then tested for normality using both the Kolmogorov-Smirnov and the Shapiro-Wilk parametric tests, which found no significant deviations from normality in all cases. Therefore we used a two-way analysis of variance (repeated measures ANOVA) test to compare the classification performance, with a Tukey post hoc test in cases of more than two treatments. An alpha of α = 0.05 was used to determine significance. A one-way ANOVA test with a Bonferroni correction was used in the cases where the effects of a treatment were tested on the classification of a single plant species.

### Generating Echoes from Spectrograms

Generating an echo from a spectrogram without phase information is impossible. In the case of our complex echoes however, the phase information is nearly random, as would be expected from a signal that is a superposition of echoes returning from many reflectors. We therefore used each column of the spectrogram as a spectrum and generated the corresponding part of the echo using a random phase. In order to prevent discontinuities when concatenating these time signals we randomly altered the phase of the frequency with the highest energy in the last created time signal such that the intensity and first derivative of its beginning matched the ones of the end of the previous time signal. This was repeated until the intensity difference was no more than 1% of the highest intensity in the last generated echo part and the first derivative of the two had the same sign. The random phase method might create problems if the spectrograms are calculated with a high overlap, because in this case the phase information in neighbouring columns is highly dependent.

To verify this method and make sure that no artefacts are created, we tested whether the random phase echoes change their class membership when analysed with our trained classifiers. For the pair apple vs. corn, for which we presented the hybrid spectrograms in the Results, we trained a classifier on original spectrograms that were created with a 10% overlap between adjacent FFT windows, and used the spectrograms of the random phase echoes as a test set. Non of the echoes changed its class after the random phase manipulation, which means that our classifiers treated the random phase echoes as representing the plant species they were supposed to imitate.
